# Female Athletes Are Half as Likely to Return to High-Risk Sports Compared With Male Athletes After Anterior Cruciate Ligament Reconstruction: An Exploration of Return-to-Sport Outcomes Between Sexes

**DOI:** 10.1177/19417381261491843

**Published:** 2026-10-12

**Authors:** Hana Marmura, Andrew Firth, Alan MJ Getgood, STABILITY Study Group, Dianne M. Bryant

**Affiliations:** † Boston Children’s Hospital, Boston, Massachusetts, United States; ‡ Western University, London, Ontario, Canada; § Fortius Clinic, London, UK; ‖ McMaster University, Hamilton, Ontario, Canada

**Keywords:** epidemiology, general sports trauma, knee, medical aspects of sports, physical therapy/rehabilitation, psychological aspects of sport

## Abstract

**Background:**

Differences in female and male athletes’ return-to-sport (RTS) outcomes after anterior cruciate ligament reconstruction (ACLR) are not well understood. The purpose of this study was to explore sex-based differences in RTS rates and predictors of RTS after ACLR for athletes participating in high-risk sports.

**Hypothesis:**

Female athletes will have significantly lower RTS rates than male athletes.

**Study Design:**

Secondary analysis of a randomized clinical trial comparing single-bundle hamstring tendon autograft ACLR with or without a lateral extra-articular tenodesis using the iliotibial band.

**Level of Evidence:**

Level 1.

**Methods:**

Data from 500 patients who underwent ACLR and participated in high-risk (jumping, pivoting, cutting) sport before injury were included in the study. Three definitions of RTS were created: return to preinjury sport and level, return to any high-risk sport at any level, and return to any sport at any level. Return rates were reported for the overall sample, and separately for male and female athletes. Multivariable logistic regression models were constructed to investigate predictors of each RTS outcome.

**Results:**

Approximately 86.5% of patients returned to any sport, whereas 76.5% returned to a high-risk sport, and only 45.5% returned to their preinjury high-risk sport and level. Male athletes reported consistently higher RTS rates than female athletes. In the final model predicting return to high-risk sport, female athletes had 53% lower odds of returning to high-risk sports compared with male athletes (odds ratio [OR], 0.47; 95% CI, 0.29 to 0.76; *P* < 0.01). For female athletes, earlier surgical intervention, higher confidence in the knee, and a stable knee were significant predictors of returning to high-risk sport.

**Conclusion:**

Female athletes are less likely to RTS after ACLR compared with their male counterparts; however, this discrepancy appears greatest for athletes returning to high-risk sports.

**Clinical Relevance:**

Female athletes in high-risk sports may be an important target population for interventions to improve RTS rates after ACLR, and psychological readiness and knee stability may be important treatment targets within this group.

Anterior cruciate ligament (ACL) tears occur primarily in young, active people, and ACL reconstruction (ACLR) is performed frequently with the objective of providing patients a stable knee that allows them to return to sport and activity.^58^ The success of ACLR is often evaluated using return to sport (RTS) as an outcome, as it represents a patient-important construct and successful completion of the rehabilitation pathway.^3,6,51,57,70^ RTS after ACLR has been defined in many ways, with operational definitions that consider different combinations of sport type, risk level, frequency, intensity, and quality of performance.^3,51^ Despite continued efforts to improve surgical techniques, rehabilitation programs, and RTS readiness tests, RTS rates are far from optimal. Estimates across studies suggest that between 19% and 100% of athletes RTS post-ACLR, depending on the definition used and population studied.^6^ The lack of consensus regarding what RTS means convolutes the literature, making it difficult to evaluate RTS interventions and to identify risk factors associated with failure to RTS. It has been suggested that RTS should be viewed on a continuum, including return to training, return to competition, and return to performance (RTP).^3,19,20^ These categories allow for important nuance in the level and intensity of sport participation after rehabilitation, but are limited in that they do not always account for changes in the type of sport an athlete returns to.

Within the group of athletes who do not RTS after ACLR, less than a quarter cite not planning to return as rationale.^42^ Combining these patients with the majority that do RTS, it appears that most patients intend to RTS after ACLR. In addition, in a study of perceived outcome importance for ACLR, patients rated return to any level of sport and return to preinjury level of sport as top 5 priorities after surgery, out of a list of 19 outcomes.^47^ Therefore, while RTS will not be the primary goal of all ACLR patients, RTS remains a key outcome of interest, and understanding how demographic and surgical characteristics influence the success of reaching different RTS definitions is paramount. Previous exploratory studies have suggested that younger age and male sex may increase the likelihood of RTS, while longer time from injury to surgery, and concomitant injuries to, and subsequent treatment of, menisci or cartilage may reduce the likelihood of RTS.^56,63^ Further investigation in other cohorts is warranted to confirm which factors may contribute to RTS type and level. Identifying predictive factors that would be known pre- or perioperatively may help clinicians shape athletes’ expectations and tailor surgery, rehabilitation, and available supports to improve RTS rates after ACLR.

An important difference in RTS rates between male and female athletes has been reported consistently in studies of patients undergoing ACLR.^7,18,41^ A systematic review published in 2023 reported that female athletes had 23% to 25% lower odds of RTS within 1-year post-ACL injury or reconstruction compared with male athletes when data was pooled from 61 studies.^18^ However, this conclusion was graded as very low certainty evidence due to heterogeneity in how RTS and patient characteristics were reported.^18^ In addition, it is not clear why female athletes would be less likely to RTS after ACLR. Female athletes are under-represented in sport medicine research,^24^ and even when both sexes are included in a study sample, data are rarely disaggregated to allow for appropriate sex-based comparisons.^39^ This limits our ability to understand both the sex-specific rates of RTS after ACLR and any factors that may influence a successful RTS for one sex but not the other.

Therefore, the purpose of this study was to explore differences in RTS data between male and female athletes who participated in high-risk sport before their ACL injury by (1) investigating results using different definitions of RTS, (2) confirming whether sex is a significant predictor of RTS, and (3) identifying whether male and female athletes exhibit different predictors of RTS.

## Methods

### Study Design and Participants

This study is a retrospective secondary analysis using data from the STABILITY 1 Study - a pragmatic, multicenter, randomized controlled trial (RCT) of young, active patients undergoing hamstring tendon autograft (HT) ACLR with or without a lateral extra-articular tenodesis (LET). Ethics approval was obtained by the Western University and local Research Ethics Boards at each participating institution, and the trial was registered at ClinicalTrials.gov (NCT02018354).^34^ Full trial methods have been published previously.^33,34^ Eligible patients were between 14 and 25 years of age, skeletally mature, with an ACL-deficient knee, and met 2 of the following 3 criteria: (1) participation in competitive pivoting (high-risk) sports, (2) presence of grade 2+ pivot shift at surgery, and (3) generalized ligamentous laxity (Beighton score of 4+) or genu recurvatum >10°.^34^ The analysis in the current study was limited to patients who participated in jumping, pivoting, and cutting sports preoperatively, to align with our objective of identifying factors predictive of RTS for athletes who participated in high-risk sports before injury. Patients were seen at 3-, 6-, 12-, and 24-months postoperatively and completed a battery of outcome measures at each visit.^33,34^

### Predictors

Predictors that are known pre- or perioperative were selected based on published literature and a priori hypotheses. We chose 7 predictors, or 8 factors, including age, sex, treatment group (i.e., HT ACLR alone or HT + LET), meniscal treatment (none, repair, excision), cartilage lesion at surgery (International Cartilage Repair Society [ICRS] cartilage lesion grade 3+), preinjury level of sport (high level = elite, varsity or competitive; low level = recreational), and time from injury to surgery.^18,56^

Previous studies have also identified that psychological factors and postoperative knee laxity are associated with RTS.^31,37,44,50,63^ While these factors are not known before, or at, surgery, we felt it was important to adjust for these confounders in sensitivity analyses. Although patients completed several questionnaires preoperatively and at each follow-up visit, a specific psychological readiness to RTS outcome measure was not used. Therefore, question 30 (q.30) on the ACL Quality of Life (ACL-QOL) questionnaire, “how much are you troubled by a lack of confidence in your knee?” was used as a proxy for this construct in our analysis.^54^ We used the score on ACL-QOL q.30, a continuous value ranging from 0 (severely troubled) to 100 (no trouble at all), from the 12-month visit unless patients had returned to sport before 12 months, whereupon the score from the visit preceding RTS (3 months or 6 months) was used. Knee laxity was assessed using the pivot-shift test at each visit by an orthopaedic surgeon blinded to surgical procedure and included as a dichotomous variable in this analysis. Patients with a pivot-shift grade of 2+ at any visit, or a grade 1 pivot shift at multiple visits, were deemed to have clinical instability, while all others were deemed stable.^33,34^

### Outcome Definitions

Patients completed questionnaires asking about their primary sport, other sport participation, and sport level pre- and postoperatively. RTS outcomes were recorded up to 2 years after ACLR. Level of sport was categorized as high (i.e., elite, varsity or competitive) or low (i.e., recreational) based on patients’ responses. We utilized 3 operational definitions of RTS using the Panther Symposium ACL Injury Return to Sport Consensus Group,^51^ and modified definitions from the RTS continuum provided by the World Congress of Sports Physical Therapy (Table 1).^3^ Sports were defined as being high-risk if they involved jumping, pivoting, or cutting. Sports defined as high-risk included but were not limited to soccer, basketball, hockey, football, rugby, downhill skiing, and volleyball. The most common examples of low-risk sports that patients participated in were running and swimming.

**Table 1.** RTS operational definitions based on criteria from the literature

RTS outcome	Operational definition
RTP	Successful return to high-risk jumping, pivoting, or cutting sport at preoperative level
RTHR	Successful return to any high-risk jumping, pivoting, or cutting sport, irrespective of level
RTA	Successful return to participation in any sport, including low-risk sports that do not require jumping, pivoting, or cutting

RTA, return to any sport; RTHR, return to high-risk sport; RTP, return to performance; RTS, return to sport.

### Statistical Analysis

An a priori sample size calculation was not performed for this post hoc analysis. Logistic regression requires a minimum of 10 events per factor to be powered adequately,^68^ and our sensitivity analysis included 10 factors; thus, 100 events were required in the least commonly occurring category for each definition of RTS.

Descriptive analyses were conducted to report RTP, return to high-risk sport (RTHR), and return to any sport (RTA) rates for the overall sample, and separately for male and female athletes, compared using a chi-square of independence. Multivariable logistic regression analyses were used to determine which factors predict RTS outcomes post-ACLR. For each RTS outcome, an initial model was constructed with all predictors included and then predictors with a *P* value >0.3 were removed sequentially using manual backward selection, until a final simplified model remained. Odds ratios (OR), 95% CI and *P* values were reported for each predictor variable in the final models. McFadden’s pseudo *R*^2^ was presented as an estimate of model fit, expressing how much the model fit improves compared with the null model, whereby values <0.10 indicate no meaningful improvement in fit from the null model and >0.20 indicate acceptable fit.^49^ Sensitivity analyses were performed for each model, by adding the confounders for confidence in the knee and postoperative knee laxity. The effect of adjusting for these factors on the final model, including updated ORs, 95% CI, and *P* values. If sex was a significant predictor in the final model, logistic regression analyses were conducted separately for male and female athletes, with the same initial set of predictor variables entered in the model, apart from sex. All assumptions for logistic regression were checked. Dependent variables were binary, and observations were independent. Multicollinearity was assessed using the variance inflation factor (VIF), with a VIF >5 indicating problematic correlation between predictors. Linearity in the logit for continuous variables was assessed visually, and predictor transformation or dichotomization was considered whether this assumption was violated. Standardized Pearson residuals were used to identify outliers, and any observations >3 were removed.

## Results

### Participants

A total of 618 patients (mean age, 19.0 ± 3.2; 51.5% female) were randomized to receive HT autograft ACLR with or without a LET in the Stability 1 RCT.^34^ From the full RCT sample, 525 patients (85%) participated in high-risk sports before their ACL injury. Four participants who experienced early graft rupture (<6 months postoperatively) were excluded as they would not have had the same ability to achieve RTS as the rest of the cohort. All other patients were included in the analysis if they had data available for all a priori predictors for the initial pre- and/or perioperative factors model, regardless of reinjury at 2-year follow-up. A total of 500 patients (82%) had the data required for the primary analysis to be included in this study (mean age, 18.8 ± 3.2; 53.2% female). The primary sport reported most was soccer, and 80% of patients participated in sport at a competitive, varsity, or elite (i.e., high) level. Significant differences between sexes were observed for age, sport type, meniscal treatment, and ICRS grade. Full patient demographics are shown in Table 2.

**Table 2.** Patient demographics, surgical characteristics, and postoperative knee stability and confidence in the knee

Participants	Overall (N = 500)	Male (n = 234)	Female (n = 266)	Male vs female *P* value
Sex				
Male	234 (46.8)			
Female	266 (53.2)			
Age, years	18.8 ± 3.2	19.7 ± 3.1	17.9 ± 3.0	<0.01*
Adolescent (14-19 years old) Young adult (20-25 years old)	313 (62.6) 187 (37.4)	117 (50.0) 117 (50.0)	196 (73.7) 70 (26.3)	<0.01*
Primary sport				<0.01*
Soccer	173 (34.6)	71 (30.3)	102 (38.3)	
Basketball	70 (14.0)	26 (11.1)	44 (16.5)	
Hockey	45 (9.0)	26 (11.1)	19 (7.1)	
Rugby	41 (8.2)	23 (9.8)	18 (6.8)	
Skiing or snowboarding	35 (7.0)	21 (9.0)	14 (5.3)	
Volleyball	33 (6.6)	5 (2.1)	28 (10.5)	
Football (American)	31 (6.2)	31 (13.2)	0 (0.0)	
Other	72 (14.4)	31 (13.2)	41 (15.4)	
Preinjury sport type and level^a^				0.46
High risk, low level	95 (19.0)	47 (20.1)	48 (18.0)	
High risk, high level	399 (79.8)	186 (79.5)	213 (80.1)	
High risk, missing level	6 (1.2)	1 (0.4)	5 (1.9)	
Graft type				0.80
HT	262 (52.4)	124 (53.0)	138 (51.9)	
HT + LET	238 (47.6)	110 (47.0)	128 (48.1)	
Meniscal treatment				<0.01*
None	186 (37.2)	79 (33.8)	107 (40.2)	
Repair	176 (35.2)	74 (31.6)	102 (38.3)	
Excision	138 (27.6)	81 (34.6)	57 (21.4)	
ICRS grade (any compartment)				0.03*
0-2	480 (96.0)	220 (94.0)	260 (97.7)	
≥3	20 (4.0)	14 (6.0)	6 (2.3)	
Months from injury to surgery	6.1 ± 11.1	6.1 ± 13.6	6.0 ± 8.4	0.95
≤3 months	215 (43.0)	99 (42.3)	116 (43.6)	0.77
>3 months	285 (57.0)	135 (57.7)	150 (56.4)	
Postoperative knee stability^b^				0.06
Stable (no pivot shift)	367 (73.4)	181 (77.4)	186 (69.9)	
Clinical instability (positive pivot)	133 (26.6)	53 (22.6)	80 (30.1)	
Postoperative confidence in knee, 0-100 (missing, n = 42)^c^	64.2 ± 28.7	66.3 ± 28.8	62.4 ± 28.6	0.15

Data are given as N (%) or mean ± SD. ACL-QOL q.30, question 30 on anterior cruciate ligament-quality of life questionnaire; HT, hamstring tendon autograft; ICRS, International Cartilage Repair Society; LET, lateral extra-articular tenodesis.

a Sport level was categorized as high (i.e., elite, varsity, or competitive) or low (i.e., recreational) based on patient survey responses.

b Patients were categorized has having clinical instability of the knee (i.e., a pivot-shift grade of ≥2 at any visit, or a grade 1 pivot shift at multiple visits) or a stable knee (i.e., no grade 2 pivot shift at any visit, or grade 1 pivot shift at a single visit) based on pivot-shift testing by a trained clinician.

c Confidence in the knee was assessed using the score on ACL-QOL q.30 - a continuous value ranging from 0 (severely troubled) to 100 (no trouble at all), from the 12-month visit unless patients had returned to sport before 12 months, whereupon the score from the visit preceding RTS (3 months or 6 months) was used.

* Significant at *P* < 0.05.

### RTS Rates

RTS data were available for 494 patients (98.8%; male: n = 231, female: n = 263) for the RTP outcome and 497 patients (99.4%; male: n = 233, female: n = 266) for the RTHR and RTA outcomes (Table 3). Approximately 86.5% of patients returned to some type of sport participation, whereas 76.5% returned to a high-risk sport, and only 45.5% returned to the same high-risk sport at the same level as they were before injury. Male athletes reported consistently higher return rates across all outcomes; however, the difference between sexes was significant only for RTHR (RTHR rates; male, 81.5%; female, 72%; *P* = 0.01).

**Table 3.** RTS rates for patients up to 2 years after ACLR

Outcome	Overall	Male	Female	*P* value
RTP	225 (45.5)	108 (46.8)	117 (44.5)	0.61
RHRS	380 (76.5)	190 (81.5)	190 (72.0)	0.01*
RTA	430 (86.5)	207 (88.8)	223 (84.5)	0.15

Data are n (%). ACLR, anterior cruciate ligament reconstruction; RTA, return to any sport; RTHR, return to high-risk sport; RTP, return to performance; RTS, return to sport.

* Significant at *P* < 0.05.

### RTS Predictors

The age and time from injury to surgery variables violated the assumption of linearity in the logit for continuous factors. Both variables were dichotomized based on thresholds established in previous literature rather than transformed with polynomials for ease of model interpretation. Adolescence is widely accepted as being up to 19 years of age.^25,29^ Therefore, participant age was dichotomized into adolescent (14 to 19 years old), and young adult (20 to 25 years old). Time from injury to surgery was dichotomized as ≤3 months, or >3 months, based on previous literature suggesting that patients who have surgery >3 months after injury were significantly less likely to return to the same or a more demanding type of sports participation.^56^ Preinjury level of sport violated the assumption of no collinearity with VIF > 70 for this predictor in all models. Therefore, preinjury level of sport was not included in the regression models. Six outliers were removed from the RTHR model and 12 outliers were removed from the RTA model. The final pared down models of predictors of RTP, RTHR, and RTA are shown in Table 4.

**Table 4.** Predictors of RTS after ACLR, using 3 operational definitions of RTS

	RTP (same high-risk sport and level)			
	Pre- and/or perioperative factors (n = 494)		Sensitivity analysis (n = 457)	
Predictor	OR (95% CI)	*P* value	OR (95% CI)	*P* value
Adolescent (14-19 years)	1.26 (0.87 to 1.84)	0.22	-	-
≤3 months injury to surgery	1.78 (1.24 to 2.56)	<0.01*	1.89 (1.28 to 2.79)	<0.01*
ICRS lesion ≥3	0.57 (0.21 to 1.55)	0.27	-	-
Confidence in knee	-	-	1.02 (1.01 to 1.03)	<0.01*
Stable knee (no pivot shift)	-	-	1.54 (0.99 to 2.39)	0.06
	Pseudo *R*^ 2^ = 0.02^a^		Pseudo *R*^ 2^ = 0.06^a^	
	RTHR			
	Pre- and/or perioperative factors (n = 491)		Sensitivity analysis (n = 454)	
Predictor	OR (95% CI)	*P* value	OR (95% CI)	*P* value
Female sex	0.51 (0.32-0.79)	<0.01*	0.47 (0.29-0.76)	<0.01*
ACLR + LET	1.31 (0.85-2.02)	0.22	-	-
≤3 months injury to surgery	2.52 (1.58-4.02)	<0.01*	2.39 (1.45-3.94)	<0.01*
ICRS lesion 3+	0.57 (0.21-1.59)	0.28	-	-
Confidence in knee	-	-	1.01 (1.00-1.02)	0.02*
Stable knee (no pivot shift)	-	-	1.75 (1.07-2.85)	0.02*
	Pseudo *R* ^2^ = 0.05^a^		Pseudo *R*^ 2^ = 0.07^a^	
	RTA			
	Pre- and/or perioperative factors (n = 486)		Sensitivity analysis (n = 444)	
Predictor	OR (95% CI)	*P* value	OR (95% CI)	*P* value
Adolescent (14-19 years)	-	-	0.62 (0.30-1.29)	0.20
Female sex	0.55 (0.31-0.98)	0.04*	0.67 (0.33-1.36)	0.27
ACL + LET	0.68 (0.39-1.19)	0.18	-	-
≤3 months injury to surgery	2.02 (1.12-3.65)	0.02*	5.40 (2.27-12.85)	<0.01*
Meniscal treatment				
Repair	0.46 (0.24-0.89)	0.02*	0.37 (0.17-0.80)	0.01*
Excision	0.76 (0.35-1.64)	0.48	1.12 (0.42-2.99)	0.83
Confidence in knee	-	-	1.02 (1.01-1.03)	<0.01*
Stable knee (no pivot shift)	-	-	1.71 (0.87-3.37)	0.12
	Pseudo *R*^ 2^ = 0.05^a^		Pseudo *R* ^2^ = 0.14^a^	

ACLR, anterior cruciate ligament reconstruction; HT, Hamstring tendon autograft; ICRS, International Cartilage Repair Society; LET, lateral extra-articular tenodesis; RTA, return to any sport; RTHR, return to high-risk sport; RTP, return to performance; RTS, return to sport.

* Significant at *P* < 0.05.

a McFadden’s Pseudo *R*^ 2^ measure of model fit improvement compared with null model.

#### Return to Performance

Sex was not a significant predictor of RTP. The only significant predictors of returning to the same preinjury high-risk sport at preinjury level were having surgery within 3 months of the injury (OR, 1.89; 95% CI, 1.28 to 2.79; *P* < 0.01) and confidence in the knee (OR, 1.02; 95% CI, 1.01 to 1.03; *P* < 0.01).

#### Return to High-Risk Sport

Sex was a significant predictor of RTHR. In the final model including only preoperative and surgical factors, female athletes were at 49% lower odds to RTHR (OR, 0.51; 95% CI, 0.32 to 0.79; *P* < 0.01) compared with male athletes. When confidence and knee stability were included in the model, female athletes were at 53% lower odds to RTHR (OR, 0.47; 95% CI, 0.29 to 0.76; *P* < 0.01) compared with male athletes. Other significant predictors of RTHR included having surgery within 3 months of the injury (OR, 2.39; 95% CI, 1.45 to 3.94; *P* < 0.01), higher confidence in the knee (OR, 1.01; 95% CI, 1.00 to 1.02; *P* = 0.02), and having a stable knee (OR, 1.75; 95% CI, 1.07 to 2.85; *P* = 0.02).

When a model was constructed including only male participants, none of the selected predictors showed a statistically significant association with RTHR. Being an adolescent (OR, 0.49; 95% CI, 0.21 to 1.10; *P* = 0.08), having surgery within 3 months of injury (OR, 1.85; 95% CI, 0.79 to 4.38; *P* = 0.16), meniscal repair (OR, 0.55; 95% CI, 0.21 to 1.43; *P* = 0.22), and confidence in the knee (OR, 1.01; 95% CI, 1.00 to 1.02; *P* = 0.16) remained in the pared down models with *P* < 0.3 (Pseudo *R*^2^ = 0.05), but 95% CIs for the estimates were imprecise. When a model including only female participants was constructed, having surgery within 3 months of the injury, higher confidence in the knee, and having a stable knee were identified as significant predictors of RTHR (Table 5).

**Table 5.** Predictors of female athletes RTHR after ACLR

	RTHR (female athletes)			
	Pre- and/or perioperative factors (n = 264)		Sensitivity analysis (n = 252)	
Predictor	OR (95% CI)	*P* value	OR (95% CI)	*P* value
≤3 months injury to surgery	3.07 (1.69-5.57)	<0.01*	2.96 (1.59-5.51)	<0.01*
ICRS lesion ≥3	0.33 (0.06-1.79)	0.20	-	-
Confidence in knee	-	-	1.01 (1.00-1.02)	0.04*
Stable knee (no pivot shift)	-	-	2.58 (1.41-4.72)	<0.01*
	Pseudo *R* ^2^ = 0.05^a^		Pseudo *R* ^2^ = 0.09^a^	

ACLR, anterior cruciate ligament reconstruction; ICRS, International Cartilage Repair Society; RTHR, return to high-risk sport.

* Significant at *P* < 0.05.

a McFadden’s Pseudo *R* ^2^ measure of model fit improvement compared with null model.

#### Return to Any Sport

Sex was a significant predictor of RTA in the initial model containing only pre and/or perioperative factors, with female athletes being at 45% lower odds of RTA compared with male athletes (OR, 0.55; 95% CI, 0.31 to 0.98; *P* = 0.04). While female sex was retained in the larger model including confidence and knee stability, it was no longer a significant predictor when controlling for these variables (OR, 0.67; 95% CI, 0.33 to 1.36; *P* = 0.27). Significant predictors of RTA included having surgery within 3 months of injury (OR, 5.40; 95% CI, 2.27 to 12.85; *P* < 0.01), having a meniscal repair (OR, 0.37 (lower odds of return); 95% CI, 0.17 to 0.80; *P* = 0.01), and higher confidence in the knee (OR, 1.02; 95% CI, 1.01 to 1.03; *P* < 0.01).

## Discussion

This study confirmed sex as an important factor related to RTS after ACLR. Female athletes reported lower RTS within 2 years of ACLR compared with male athletes participating in similar high-risk jumping/cutting/pivoting sports. The most important finding of this study was that female athletes were approximately half as likely to RTHR compared with male athletes, with 95% CI indicating female athletes had 21% to 71% lower odds of RTHR. When study data was disaggregated by sex, the RTHR models were not similar for male and female athletes. None of the selected variables (i.e., age group, treatment group, meniscal treatment, cartilage lesion at surgery, time from injury to surgery, confidence in the knee, knee stability) were significant predictors of RTHR for male athletes. However, having surgery within 3 months of injury, higher confidence in the knee, and having a stable knee (i.e., no pivot shift) were significant predictors of RTHR for female athletes.

Having ACLR within the first 3 months after injury, confidence in the knee and knee stability were consistent predictors of RTS across models. Having earlier surgery (≤3 months from ACL rupture) was associated with 1.9 to 5.4 times higher likelihood of RTS. These results mirror findings from a cohort study of 251 ACLR patients published in 2022 by Muller et al^56^ showing that patients were approximately 70% less likely to return to preinjury sport if their ACLR surgery occurred >3 months after surgery (OR, 0.31; 95% CI, 0.17 to 0.58; *P* < 0.01). The optimal time between ACL injury and ACLR is heavily debated and varying definitions of early versus delayed surgery make study results difficult to compare (i.e., delayed surgery has been defined as early as 1-month and as late as 12-months postoperatively).^56,57^ Delayed surgery has been associated with more intra-articular pathology in patients after ACL injury,^2,16,52,71^ which may impact RTS rates negatively. It is also possible that the addition of time before surgery lengthens the recovery and/or rehabilitation process to a point where RTS is less appealing, feasible, or impactful for patients, especially for young athletes who may have RTS goals centered around high school or college years and/or seasons. The potential biases (i.e., sex, gender, sport, accessibility, anatomy, physical function, psychological factors, etc) that may affect what type of patients receive early surgery are unclear and important topics for further research.

Having a stable knee was similarly associated with a 1.5 to 2.6 higher likelihood of RTS. Importantly, in this study patients were categorized as having a stable knee if they did not meet the criteria for clinical laxity - a grade 2 pivot-shift test at any follow-up visit or a grade 1 pivot-shift test at >1 follow-up visit. This definition encompasses a wider range of patients whose sport-specific activities may be affected by residual rotational laxity after ACLR. Knee stability has been established previously as a factor related significantly to RTS in our sample, with greater proportions of patients with a stable knee being able to return to increasingly demanding levels of sport.^63^ This idea is supported by a study of 132 ACLR patients >16 years old reporting that less anterior tibial displacement using the KT-1000 arthrometer measurement was a significant predictor of 2-year RTS.^31^

The RTS analyses in this study indicated consistently that every 1-point increase in the question “how much are you troubled by a lack of confidence in your knee?” on a scale from 0 to 100 would be associated with a 1% to 2% increase in likelihood of RTS, meaning a 10% increase in confidence would be associated with a 10% to 20% increased likelihood of RTS. This pattern supports a body of previous literature suggesting that psychological factors provide important contributions to whether an athlete will RTS successfully post-ACLR.^4,8,21,37,44^ Rates of returning to high level competitive sport remain low despite high rates of acceptable knee-related function, suggesting that other variables such as psychological and cognitive factors may contribute more strongly to RTS decisions.^9,12^ This premise is confirmed further by the consistent finding that fear of reinjury, movement, or knee symptoms is the number one reason for athletes not returning to sport or reducing their sport participation,^5,9,12,61,63^ and that knee self-efficacy (i.e., a person’s belief in their ability to perform activities involving their knee) is associated with successful return to sport.^12,13,35^ In wanting to account for psychological considerations, we chose ACL-QOL q.30, which asks about patients’ confidence in their operative knee. This question is likely highly related the psychological construct of knee self-efficacy and corroborates these previous findings.

The only variable that showed significant a difference between male and female athletes in our descriptive demographics analysis but was not included in regression modeling was primary sport. This variable was not included as a predictor due to the wide variation of sports reported with insufficient categorical numbers across all sports and sexes for an appropriate analysis. Given the sample was already restricted to those participating in high risk cutting and pivoting sports before their ACL rupture, we did not feel it would be appropriate to combine sport types. However, it is possible that some of the sex-based differences observed in this study are due to sport-specific factors. Post hoc analyses revealed that the difference in sport participation between male and female athletes was driven by the proportion of patients reporting American football (male, 13.2%; female, 0%), and volleyball (male, 2.1%; female, 10.5%) as their primary sport. There is a dearth of previous literature comparing RTS rates stratified by sport type within a single study sample. However, individual studies show similar RTS rates across high-risk sports such as soccer, basketball, hockey, and football.^17,26,27,30,36,46,48^ One systematic review of elite and professional sports identified lower rates of return to play (defined as playing in ≥1 game after ACLR in the same sport) in Australian rules football and American football compared with soccer.^27^ This would not support the idea that male athletes in our sample have higher RTS rates based on sport-specific context; however, volleyball was not included in the referenced review.

The models for RTHR built with disaggregated data suggest that earlier ACLR, confidence in the knee, and knee stability may be factors specifically relevant to female athletes post-ACLR, and it is possible that female patients were driving the significance of these factors in the overall models. Female athletes commonly report worse psychological readiness to RTS outcomes (i.e., higher levels of fear, lower confidence and self-efficacy) as compared with male athletes,^41,43,53,72^ which may explain why confidence in the knee was a significant predictor of RTHR for female athletes, but not for their male counterparts. People who do not RTS may also exhibit a lower level of risk appraisal, which some argue as protective rather than maladaptive.^65^ Younger patients and those with higher confidence tend to perceive RTS post-ACLR as lower risk and be more likely to return,^59,64^ aligning with the results in the current study that younger age and higher confidence were RTHR predictors in the female group. While sex-based differences in risk appraisal have yet to be confirmed in ACL specific samples,^59,64^ large-scale psychological and epidemiological studies have indicated that women report a significantly lower willingness to take risks than men,^28^ and that men are at 20% greater risk of injury due to lifestyle activities compared with women.^66^ It is possible that these systematic sex-based differences in attitudes and cognitive appraisals leads to a lower RTS rate post-ACLR for female athletes.

In the female-specific RTHR model, patients who had surgery within 3 months of injury, and patients with a stable knee, were almost 3 times more likely to RTHR. The ORs in the female specific model for these factors were both larger than those in almost all other overall models. While female patients in this study did have a higher proportion of patients with clinical instability compared with their male counterparts (30% versus 23%; *P* = 0.06), the between-group difference was not significant for knee stability or time to surgery. Therefore, it may be true that knee (in)stability and timing of surgery affect female athletes differently, potentially through cognitive mechanisms linked to decreased psychological readiness to RTS in female athletes, or difficulty achieving other functional outcomes relevant to a successful return.

Across a variety of sporting contexts, boys are about 4 times more likely to participate in organized sport during adolescence than girls,^26^ and girls are significantly more likely to drop out of sport during this time.^29,55^ Reasons for sport drop-out include lack of enjoyment, perceptions of competence, social pressures, competing priorities, and physical factors (i.e., maturation and injury).^25,55^ Therefore, it is likely that experiencing a traumatic injury such as an ACL rupture would exacerbate this phenomenon. Considering the age range of our sample (i.e., 14 to 25), and the fact that there was a significantly higher proportion of adolescent female athletes (74%) compared with male athletes (50%), female athletes would be making RTS decisions during or just after adolescence, when sport cessation rates are highest. The sex-based differences observed in youth sport attrition,^29,55^ and difference in age groups in the current sample may help explain the differences in RTHR rates observed between male and female patients post-ACLR. Overall, it is also likely that patients in the young adult age group (20 to 25 years old) may be aging out of sport or naturally changing their type and level of sport participation due to life and environmental factors (i.e., transitions between high school, college, workforce, etc).

We operationalized RTS in 3 ways to account for a continuum of potential outcomes, including that patients may switch their primary sport to a “lower risk” option (RTA) or may return to their preinjury sport at a lower level (RTHR). As expected, the RTP outcome of returning to a high-risk sport at preoperative level showed the lowest return rate at 45.5%, whereas the most liberal outcome of RTA had almost double the RTP return rate at 86.5%. These RTS rates reflect the estimates reported in the literature ranging from 40% to 80%^6,7,9^ and emphasize the need for clear reporting of standardized RTS outcomes in sport medicine research.

The estimates of model fit were poor across all models. Model fit improved with the addition of the 1-year postoperative outcomes but did not reach our threshold of acceptable fit (i.e., pseudo *R*^2^ ≥ 0.20). These results highlight the difficulty associated with predicting RTS outcomes in young athletes, especially when only considering factors known preoperatively or at the time of surgery. It could be argued that functional outcomes such as strength and hop testing results used in RTS testing batteries might be better predictors of RTS outcomes. However, a study by Rezansoff et al^63^ in 2023 using the same RCT population as the current study found there was little difference in patient-reported and functional outcomes between patients who returned to various sport types and levels. Previous studies have similarly indicated that functional outcomes and RTS testing outcomes are not strong predictors of RTS post-ACLR.^12,44^

### Clinical Implications

Improving RTS rates for those who want to RTS after ACLR means that more people will experience the physical and psychological benefits of regular physical activity and sport participation. Altogether, the results of this study suggest female athletes are at greatest risk of failing to RTS after ACLR when compared with male athletes playing similar sports (i.e., high-risk jumping/cutting/pivoting sports). As such, rehabilitation programs with specific considerations for this population, and increased access to care and support for women’s sport organizations may be beneficial. Currently, RTS protocols are not well developed, rigorously tested, or standardized for female athletes and sex-specific protocols are recommended.^69^ In addition, psychological factors appear to be a primary treatment target for female athletes attempting to RTS. Assessment of psychological readiness to RTS is now commonly recommended as part of RTS testing; however, the integration of intervention strategies to target maladaptive psychological responses is not yet part of regular practice. Preliminary evidence suggests that psychologically informed rehabilitation strategies including imagery, mindfulness meditation, relaxation, improving coping skills, positive self-talk, and goal setting may improve psychological responses to ACL injury and ACLR.^10,11,23,40^ Finally, surgical timing and knee stability appear particularly impactful to female RTS rates. Therefore, surgical decisions related to timing and technique (i.e., graft type and additional procedures) should be made to improve the chances of avoiding residual postoperative knee laxity in female patients. A systematic review by Paterno et al^62^ reported that female patients who had an ACLR with a hamstring autograft experienced greater anterior-posterior laxity than male patients who had a hamstring autograft, or female and male patients who had a bone patellar tendon bone autograft. The addition of a lateral procedure, such as a LET or anterolateral ligament reconstruction, to ACLR has been endorsed as significantly improving knee stability compared with isolated procedures,^14,15,34,38,45^ and may be an important consideration for female athletes attempting to RTHR.^32^

### Limitations

Being a retrospective secondary analysis of an RCT, this study comes with limitations. First, the population of the study is narrow, including only patients 14 to 25 years of age, deemed to be at high risk of reinjury, and who participated in high-risk sports before injury. While the results of this study provide important information about a population that may be more likely to experience a secondary injury and may benefit most from improved RTS testing and rehabilitation interventions, the results cannot be applied to all people with an ACL injury. In addition, the questionnaires included in the original RCT did not include a measure to specifically assess psychological readiness to RTS, and RTS information recorded was limited. A validated assessment of psychological responses to injury such as the ACL Return to Sport after Injury Scale (ACL-RSI) and a more detailed questionnaire about sport type, frequency, intensity, and objective and subjective measures of performance quality would be helpful in future RTS research. Importantly, we cannot be sure that all athletes in the current sample were striving for our outcomes of interest (i.e., various levels of RTS). Changing patient priorities, risk appraisal, and decision making will certainly influence RTS outcomes and are difficult to account for in RTS studies. Future studies should collect patient intentions and goals, not just at baseline but longitudinally, to identify those who did not attempt to RTS, and perform appropriate sensitivity analyses of RTS outcomes without these athletes. Finally, only sex-based differences were explored in this analysis, and it is likely that many of the differences observed between male and female athletes may be due to differences related to gendered environments in sporting, medical, rehabilitation, and social contexts, rather than specifically linked to characteristics of biological sex (i.e., anatomy, genetics, hormones, etc).^1,22,60,67^ However, we cannot draw these conclusions based on the current study.

## Conclusion

Female athletes participating in jumping, cutting, and pivoting sports are less likely to RTS after ACLR compared with their male counterparts; however, this discrepancy is most stark for athletes RTHR. Female athletes in high-risk sports may be an important target population for intervention to improve RTS rates after ACLR, and psychological readiness and knee stability may be important treatment targets within this group.
